# A cheap and open HIV viral load technique applicable in routine analysis in a resource limited setting with a wide HIV genetic diversity

**DOI:** 10.1186/s12985-017-0893-3

**Published:** 2017-11-14

**Authors:** Elodie Téclaire Ngo-Malabo, Paul Alain Ngoupo T., Martin Zekeng, Valérie Ngono, Laure Ngono, Serge Alain Sadeuh-Mba, Richard Njouom, Anfumbom Kfutwah

**Affiliations:** Virology Department, Centre Pasteur of Cameroon, Po Box 1274, Yaounde, Cameroon

**Keywords:** HIV viral load quantification, Serotyping- group indeterminate, Resource limited setting, Generic HIV viral load, Abbott real-time HIV-1

## Abstract

**Background:**

HIV infection in Cameroon is characterized by a great viral diversity with all HIV-1 groups (M, N, O, and P) and HIV-2 in circulation. HIV group determination is very important if tailored viral load analysis and treatments are to be applied. In our laboratory, HIV viral load is carried out using two platforms; Biocentric and Abbott depending on the HIV group identified. Biocentric which quantifies HIV-1 group M is a cheap and open system useful in resource limited settings. The objective of this study was to compare the viral load analyses of serologically group-indeterminate HIV samples using the two platforms with the view of reducing cost.

**Methods:**

Consecutive samples received between March and May 2014, and between August and September 2014 in our laboratory for HIV viral load analysis were included. All these samples were analyzed for their HIV groups using an in-house ELISA serotyping test. All HIV-1 group M samples were quantified using the Biocentric test while all other known atypical samples (HIV-1 groups N, O and P) were analyzed using the Abbott technique. HIV group-indeterminate samples (by serotyping) were quantified with both techniques.

**Results:**

Among the 6355 plasma samples received, HIV-1 group M was identified in 6026 (94.82%) cases; HIV-1 group O, in 20 (0.31%); HIV-1 group M + O, in 3 (0.05%) and HIV-2, in 3 (0.05%) case. HIV-group indeterminate samples represented about 4.76% (303/6355) and only 231 of them were available for analysis by Abbott Real-Time HIV-1 and Generic HIV Viral Load techniques. Results showed that 188 (81.39%) samples had undetectable viral load in both techniques. All the detectable samples showed high viral load, with a mean of 4.5 log copies/ml (range 2.1–6.5) for Abbott Real-Time and 4.5 log copies/ml (range 2–6.4) for Generic HIV Viral Load. The mean viral load difference between the two techniques was 0.03 log_10_ copies/ml and a good correlation was obtained (*r* ^*2*^ = 0.89; *P < 0.001*).

**Conclusion:**

Our results suggest that cheaper and open techniques such as Biocentric could be useful alternatives for HIV viral load follow-up quantification in resource limited settings like Cameroon; even with its high viral diversity.

## Background

Human immunodeficiency virus (HIV) infection is a major public health problem in the world, particularly in sub-Saharan Africa where the majority of patients live. An outstanding characteristic of the virus is its genetic variability which has been attributed to high rates of mutation [1], recombination and viral turnover [2]. To date, HIV is divided into two types: HIV-1 and HIV-2. HIV-1 has been subdivided into four phylogenetically distinct groups: M for major (or main), O for outlier, N for non-M/non-O (or new) and P [3, 4], while HIV-2 is subdivided into nine groups: A-I [5]. According to the demographic health survey (DHS) 2011, the seroprevalence of HIV is estimated at 4.3% in Cameroon [6]. This infection is marked by a great genetic diversity with the co-circulation of all types and groups (HIV-1 M-P and HIV-2). This diversity has been shown to impact on the diagnosis (possibility of false negative results) [7], on treatment; (some studies described that HIV-1 O were naturally resistant to the non-nucleoside reverse transcriptase inhibitor because of the presence of Y181C mutation in the RT gene [8]) and on follow-up of patients. Therefore, diagnostic techniques (screening and molecular biology), follow-up and treatment options are really dependent on the type and/or group of HIV. HIV-1 O for example has a broader diversity than HIV-1 M [9] and causes great difficulty for diagnosis. The high genetic diversity of HIV-1 also has a major impact on the plasma quantification of HIV-1 RNA [10, 11]. HIV group determination is very important if tailored viral load analysis and treatments are to be applied.

In the biological follow-up of HIV infected patients, plasma (RNA) viral load is the key parameter currently recommended by WHO. In fact it is a good marker of therapeutic adherence, disease progression and treatment efficacy. In addition, according to recent WHO’s recommendations, it will be used as the main therapeutic follow-up parameter rather than CD4 counts.

For HIV plasma viral load analyses, the Virology Department of Centre Pasteur of Cameroon uses two platforms: Generic HIV Viral Load assay (Biocentric, Bandol, France) and Abbott Real-Time HIV-1 assay (Abbott Molecular, Wiesbaden, Germany). The techniques choice is based on the HIV-1 group harbored by the patient. Generic HIV Viral Load assay, used for the quantification of HIV-1 group M is a cheap and open system whose usefulness has been shown in resource limited countries like Cameroon. It has also been shown to be applicable in the context of high viral diversity among HIV-1 group M. Abbott Real-Time HIV-1 assay is broader in terms of range of HIV groups covered [10], however because of unaffordable cost of analysis, this test is less implemented in resource limited settings. In order to choose an adequate and cheap HIV viral load technique in our laboratory for each sample, HIV types/groups determination is performed routinely using an in-house ELISA assay as previously described [12]. In spite of the systematic application of this discriminatory test, the types/groups of some samples remain indeterminate. The objective of this study was to compare the viral loads of serologically indeterminate HIV samples using two techniques: the Generic HIV Viral Load assay (Biocentric, Bandol France) and the Abbott Real-Time HIV-1 assay (Abbott Molecular, Wiesbaden Germany) with the view of reducing cost in such genetically divergent viral populations.

## Methods

### Description of the study site

The Centre Pasteur of Cameroon (CPC) is one of the reference HIV laboratories in Cameroon. It plays a central role in evaluating HIV tests intended to be used for routine analyses in Cameroon. As part of this role, the CPC determined the utility a cheaper test, the Biocentric method for HIV serological indeterminate samples. All the data were collected in the Virology service from patients who requested HIV viral load analyses at CPC. Two platforms are used for viral load quantification selected according to HIV serotyping results: Biocentric is specifically dedicated for HIV-1 group M quantification while Abbott is used for other HIV-1 variants.

### Serotyping

Between March and May 2014, and between August and September 2014, 6355 consecutive samples were registered in the Virology laboratory of Centre Pasteur of Cameroon for viral load analysis. As a routine test, prior to all viral load analysis in our laboratory, HIV-group serotyping was performed on all these samples using an in-house ELISA as previously described [12]. Briefly, this test uses HIV peptides of the V3 loop and gp41/36 regions of HIV-1 groups M, N, O and P as well as HIV-2 peptides. A first test, “two peptides” format, explores the V3 region of HIV-1 groups M (HIV-1 M) and O (HIV-1 O). Negative samples in the “two peptides” assay were re-tested in a second ELISA known as the “ten peptides” format using peptides mapping the gp41/36 region of HIV-1 groups M, O, N and HIV-2/SIVsm on one hand and V3 peptides of HIV-1 groups M, O (subgroup H and consensus), N, P, and HIV-2/SIVsm on the other hand.

### Plasma HIV RNA quantification

All HIV-1 M samples identified by serotyping were quantified by the Generic HIV Viral Load assay (Biocentric, Bandol-France), which targets a well-conserved LTR region of HIV-1 [13]. Non-M samples (HIV-1 N, O and P) identified by serotyping were quantified by Abbott Real-Time HIV-1 assay (Abbott molecular, Wiesbaden-Germany), which targets a highly conserved integrase-coding region of the pol gene [14]. For the purpose of this study, HIV group-indeterminate samples (by serotyping) were quantified with both techniques.

- **Generic HIV Viral Load assay (Biocentric Bandol-France)**

RNA was extracted manually from 1 mL of all HIV-1 group M plasma samples using QIAmp viral RNA mini kit (QIAGEN, Courtaboeuf, France) according to the manufacturer instructions. Purified RNA was eluted in 60 μL of molecular grade water. A volume of 10 μL of RNA extracts was therefore used for quantification with the Generic HIV Viral Load assay (Biocentric, Bandol-France) as originally described by Rouet and collaborators in the ANRS HIV quantification working group [13] and previously reported by our team [15]. The cycling conditions consisted of 50 °C for 10 min and 95 °C for 5 min, followed by 50 cycles of 95 °C for 15 s and 60 °C for 1 min. Amplification and data acquisition were carried out using the ABI Prism 7300 Sequence Detection System (Applied Biosystems) and the detection cut-off value was 60 HIV-1 RNA copies/mL.

b. **Abbott Real-Time HIV-1 assay (Abbott molecular, Wiesbaden-Germany)**

This assay was performed on samples that tested HIV-1 Group O or N by serotyping. The test was run according to the manufacturer’s instructions. Briefly, HIV-1 RNA was extracted from 0.6 mL of plasma sample, using the Abbott m2000sp nucleic acid extraction system. 50 μL of purified RNA was subsequently mixed with 50 μL of Master mix and run on the m2000rt Real-Time PCR system [14]. The detection cut-off value was 40 HIV-1 RNA copies/mL.

### Molecular characterization

For the purpose of this study, samples that were serologically HIV type/group-indeterminate were quantified by both the Abbott Real-Time HIV-1 and Generic HIV Viral Load assays. Samples showing detectable viral loads were further characterized using molecular tests.

Molecular tests were performed using an RT-nested PCR targeting the pol gene (Integrase) of HIV-1 group M and O as previously described [16]. RNA extracts originating from samples that were detectable by only one of the quantification techniques (Generic HIV Viral Load and Abbott HIV-1 viral load assays) were further analyzed using an RT-nested PCR targeting the Gp41 region of the Env gene as previously described [17]. This PCR uses primers with a broad specificity and can amplify HIV-1 (M, O, and N) as well as Simian Immunodeficiency Viruses from Chimpanzees (SVIcpz). Primers were designed on the basis of conserved gp41 (gpM–Z) regions. The outer primers gp40F1 and gp41R1 and the inner primers gp46F2 and gp48R2 were used with the PCR conditions previously described [17].

### Sequence and phylogenetic analyses

PCR products were sequenced by the Sanger method using BigDye® Terminator Cycle Sequencing Ready Reaction v3.1 kit (Applied Biosystems). Sequences were aligned by CLUSTALW and phylogenetic trees were inferred using a Kimura two-parameter substitution model and the neighbor-joining method with 1000 bootstrapped data sets implemented with the MEGA6.06 software [18].

### Statistical analysis

We performed the correlation coefficient of Pearson to observe association between HIV-1 viral loads obtained with the two techniques. Furthermore, the statistical tests of Karle Wallis were implemented to compare the value of correlation with 0. All the statistical analyses were performed using R software version 2.15.

## Results

### Serotyping

Among the 6355 plasma samples received, HIV-1 group M was identified in 6026 (94.82%) cases, HIV-1 group O in 20 (0.31%), HIV-1 group M + O in 3 (0.05%) and HIV-2 in 3 (0.05%) cases. HIV-group indeterminate samples represented about 4.76% (303/6355) of all samples received.

### Abbott real-time HIV-1 and generic HIV viral load assays of HIV group indeterminate samples

Because of the limitation of the volume of some plasma samples, only 231 of the 303 types/groups indeterminate samples were available and could be analyzed by Abbott Real-Time HIV-1 and Generic HIV Viral Load techniques. The results showed that 188 (81.39%) of these samples showed undetectable viral load using both techniques. All the remaining 43 samples were detectable with Abbott Real-Time HIV-1 while 40 of them were also detectable with the Generic HIV Viral Load (Table 1). All detectable samples in both techniques showed high viral load, with a mean of 4.5 log copies/ml (range 2.1–6.5) and 4.5 log copies/ml (range 2–6.4) for Abbott Real-Time and Generic HIV Viral Load, respectively.

**Table 1:** Viral load of HIV serotyping indeterminate samples

		Abbott Real-Time HIV-1		
		Detectable	Undetectable	Total (%)
Generic HIV Viral Load	Detectable	40	0	40 (17.32)
	Undetectable	**3**	188	191 (82.68)
	Total (%)	43 (18.61)	188 (81.39)	231

Plasma viral load results of the 231 HIV group indeterminate samples obtained with Abbott Real-Time HIV-1 and Generic HIV Viral Load are shown in the table. The number of samples that were detectable by one of the two assays is shown in bold

**Fig. 1:**
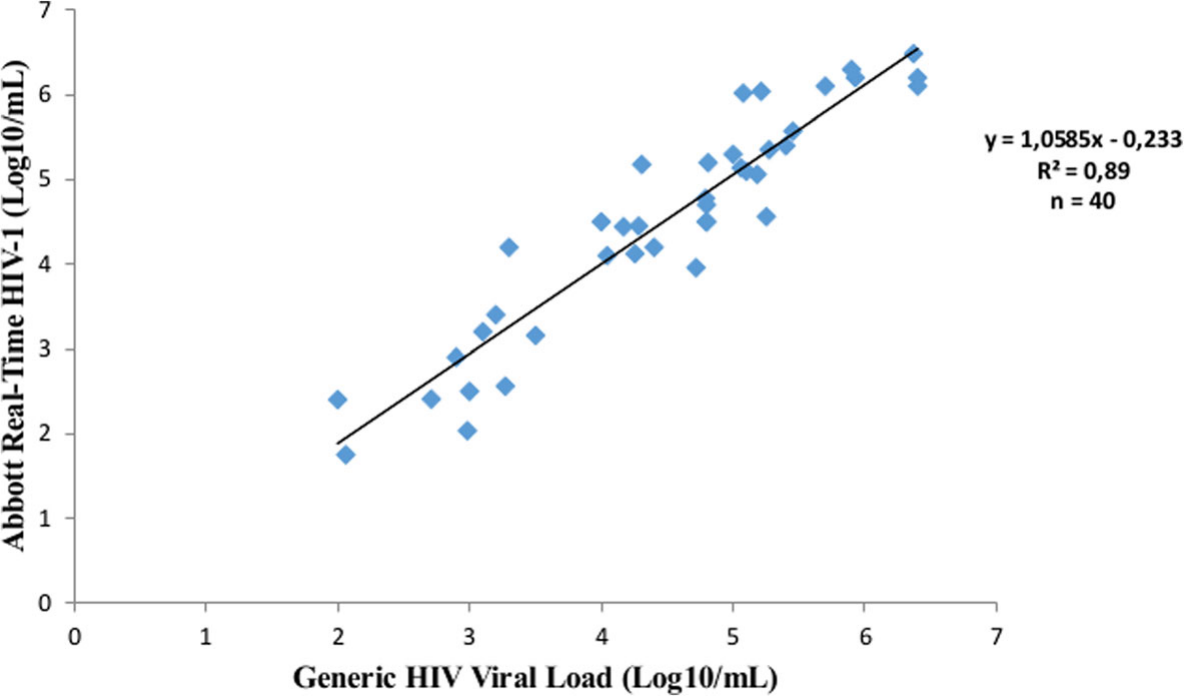
Correlation between Generic HIV viral load and Abbott Real-Time HIV-1 assays. Using the samples (*n = 40)* detected with both techniques, Viral loads obtained with Abbott Real-Time HIV-1 (Y-axis) assays were plotted against values of Generic HIV viral load (X-axis). The solid line represents the fitted regression and the Pearson correlation coefficient *r* ^*2*^ = 0.89 (*P < 0.001*) confirms the good correlation between both techniques

The mean viral load difference between the two techniques was 0.03 log_10_ copies/ml which was not significantly different. Importantly, the difference between both assays did not increase with low or high viral loads. A good correlation was found between the results of both assays (Pearson correlation coefficient *r* ^*2*^ = 0.89; *P < 0.001*) as shown on Fig. 1.

### Molecular characterization

**Fig. 2:**
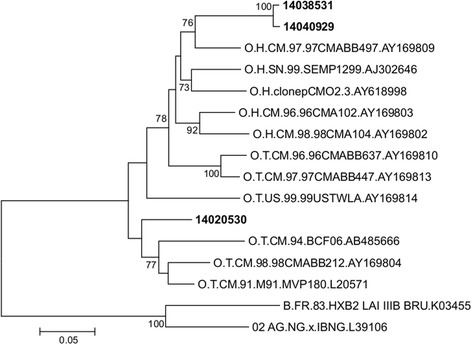
Phylogenetic characterization of the discordant samples between Abbott. Real-Time HIV-1 and Generic HIV Viral Load. Phylogenetic tree based on unambiguously aligned nucleotides from the Gp41 region (386 bp). Sequences from the three samples with discordant results on Abbott and Biocentric platforms are shown in bold. The subgroup of reference sequences representative of HIV-1 group O is indicated in the name of the sequence (H = head and T = tail). Two HIV-1 group M sequences, B.FR.83.HXB2 LAI IIIB BRU.K03455 and 02 AG.NG.x.IBNG.L39106 were used as outgroups. The analysis was performed as described in Methods section (Sequence analysis)

Molecular characterization of HIV type/group indeterminate samples showed that all forty samples detectable by both techniques were HIV-1 group M while the three discordant samples detected only by the Abbott Real-Time HIV-1 technique were HIV-1 group O. Further phylogenetic analysis of the sequences of the Gp41 region derived from the three HIV-1 group O samples, showed that two of them belonged to HIV-1 group O subgroup H while the last one was a member of the subgroup T (Fig. 2).

## Discusion

Plasma (RNA) viral load is the key parameter currently recommended by WHO for the biological follow-up of HIV infected patients because it is a good marker of therapeutic adherence, disease progression and treatment efficacy. In our laboratory, two platforms are used for viral load quantification selected according to HIV serotyping results: Generic HIV Viral Load (Biocentric) is specifically dedicated for HIV-1 group M quantification and Abbott Real-Time (Abbott) for other HIV-1 variants. Serotyping used for the discrimination of different HIV variants (HIV-1 groups M, N, O and P and HIV-2), is performed using two sets of peptides: the gp41/36 and the V3 specific for each HIV type/group. Data have shown that the gp41/36 peptides have an excellent sensitivity while the V3 peptides have an excellent specificity for the corresponding HIV-1 group, especially the group M peptides [19]. Simon and collaborators demonstrated that the V3 discrimination component of the test clearly identified all the group M samples and there was no V3 reactivity other than that directed to the peptides mapping this group. Serotyping could therefore be systematically performed as complementary test for all HIV-positive samples because this will help for the choice of appropriate treatment option as well as the platform to be used for viral load. Thus, samples classified as HIV-1 group M in our study (6026 out of 6355 samples received) were correctly quantified by Generic HIV Viral Load (Biocentric).

Our serological data confirmed the circulation of HIV-1 (groups M, N and O), HIV-2, as well as HIV-1 M + O dual infections. These data confirm the great genetic diversity of HIV in Cameroon [20–22]; with HIV-1 M representing the broad majority of HIV in circulation. It is known that HIV-1 M is the pandemic form of HIV because it is distributed worldwide, while other variants are mostly found in Africa, especially in Cameroon [23]. Our results showed that 0.3% of samples were HIV-1 group O, and this is consistent with the actual epidemiology of HIV-1 group O whose prevalence is estimated at approximately 0.4–1% of all HIV infections in Cameroon [20–22]. Among the 231 HIV type/group indeterminate samples analyzed with both Generic HIV Viral Load and Abbott Real-Time assays, as high as 81.39% had undetectable viral load in both techniques. This could be due to the fact that most of patients received in our laboratory for HIV viral load are on Combined Antiretroviral Therapy; and according to the WHO 2013 recommendations which are used in the country, viral load is performed six months after treatment initiation. These undetectable VL results indicate the absence of plasma HIV-1 RNA and reveal expected treatment efficacy for the majority of the patients received. It has been demonstrated that treatment can drop viral load and antibody titers can subsequently decrease leading to indeterminate serological tests [24] as observed with indeterminate results in serotyping. However, PCR could be used to confirm HIV status in such cases; we previously described seronegative results with positive DNA PCR in children who started treatment early [25].

We found a good concordance (98.7%) between both techniques, and the majority of samples found to be detectable with Abbott Real-time were also detectable with Generic HIV Viral Load, with a good correlation between viral load values (Pearson correlation coefficient *r* ^*2*^ = 0.89; *P < 0.001*). This is consistent with results obtained by Rouet and collaborators who described the performance of the ANRS (Agence Nationale de Recherche sur le SIDA) second-generation long terminal repeat-based real-time RT-PCR test, Generic HIV Viral Load, for the quantification of HIV in a context of high HIV genetic diversity [13]. The molecular characterization showed that all 40 samples efficiently detected by both techniques were HIV-1 group M, whereas, the three samples refractory to Generic HIV Viral Load were HIV-1 group O. This result can be explained by the fact that Generic HIV Viral Load has been designed for the quantification of HIV-1 group M and has been shown to inaccurately quantify certain strains of HIV-1 group O [13]. In the phylogenetic analysis, two of HIV-1 group O sequences were closely related and supported by a high bootstrap value (Fig. 2). Further analyses were performed to investigate a potential transmission pair. These two sequences displayed a 97.43% identities and they were obtained from two women from different cities. Also, the patients were received at different date with no epidemiological link. Altogether, these results suggested that there is no transmission pair even if the two sequences are more related to each other than to other HIV-1 group O sequences.

Even though viral load has become the main virological marker for detecting treatment failure, this test is not affordable in many countries especially in the resource limited settings. Because of inadequate laboratory capacity and high cost of equipment, viral loads are performed in the reference laboratory; samples have to be collected in the field and shipped to reference laboratory for testing. In this context, logistic barriers (plasma separation, storage and shipping of specimens) are major challenges in the availability of HIV viral load in peripheral regions. Therefore, there is an urgent need in the development of less costly methods such as viral load pooling, viral load using DBS as well as point of care to monitor patients receiving antiretroviral therapy. These methods could be helpful for the sustainability of the monitoring of patients; however, such strategies should to be validated in each region/country in order to take into consideration the specificity of the designated area.

Nonetheless, our study has some limitations because the presence of non HIV-1 variants in the 188 samples with HIV type/group indeterminate results with serotyping and undetectable viral load on both Abbott Real-time and Generic HIV Viral Load cannot be ruled out. Since these viral load assays could not amplify non HIV-1 variants, nested-PCR for HIV-2 and SIV should be performed on these samples.

## Conclusion

In conclusion, our results suggest that a cheaper and open technique such as Generic HIV Viral Load (Biocentric) could be a reliable alternative for HIV viral load follow-up quantification in the settings marked by a high viral diversity, like Cameroon. In such settings, molecular characterization could therefore be performed on samples originating from patients with immuno-virological or immuno-clinical discordances, in order to detect potential non-M HIV variants. Since the price of Abbott Real-Time assy is twice the price of the Generic assay in Cameroon, using this Biocentric platform will save 450,000 US dollars at the end of the year.

## Abbreviations

cART: Combined Antiretroviral Therapy
CPC: Centre Pasteur du Cameroun
DHS: Demographic Health Survey
HIV: Human Immunodeficiency Virus
PCR: Polymerase Chain Reaction
VL: Viral Load
WHO: World Health Organization
